# The most 5′ truncating homozygous mutation of *WNT1* in siblings with osteogenesis imperfecta with a variable degree of brain anomalies: a case report

**DOI:** 10.1186/s12881-018-0639-0

**Published:** 2018-07-16

**Authors:** Chulaluck Kuptanon, Chalurmpon Srichomthong, Apiruk Sangsin, Dool Kovitvanitcha, Kanya Suphapeetiporn, Vorasuk Shotelersuk

**Affiliations:** 1 Department of Pediatrics, Queen Sirikit National Institute of Child Health, Bangkok, 10400 Thailand; 2 Center of Excellence for Medical Genomics, Department of Pediatrics, Faculty of Medicine, Chulalongkorn University, Bangkok, 10330 Thailand; 3 Excellence Center for Medical Genetics, the Thai Red Cross Society, King Chulalongkorn Memorial Hospital, Bangkok, 10330 Thailand; 4 Department of Orthopaedics, Faculty of Medicine, Chiang Mai University, Chiang Mai, 50200 Thailand; 5 Department of Orthopedics, Queen Sirikit National Institute of Child Health, Bangkok, 10400 Thailand; 6 Division of Medical Genetics and Metabolism, Department of Pediatrics, Faculty of Medicine, Chulalongkorn University, Sor Kor Building 11th floor, Bangkok, 10330 Thailand

**Keywords:** Brain anomalies, Osteogenesis imperfecta, WNT1, Mutation, Phenotype, Case report

## Abstract

**Background:**

*WNT1* mutations cause bone fragility as well as brain anomalies. There are some reported cases of *WNT1* mutations with normal cognition. Genotype and phenotype correlation of *WNT1* mutations has not been established.

**Case presentation:**

Here we present two female siblings with osteogenesis imperfecta (OI) born to a consanguineous couple. Both sustained severe bone deformities. However, only the younger had severe brain anomalies resulting in an early death from pneumonia, while the older had normal intellectual development. Next generation sequencing showed a homozygous mutation, c.6delG, p.Leu3Serfs*36 in *WNT1*. To our knowledge, it is the most 5′ truncating mutation to date*.*

**Conclusion:**

This report emphasizes the intrafamilial variability of brain anomalies found in this OI type and suggests that WNT1 may not be necessary for normal human cognitive development.

## Background

Osteogenesis imperfecta (OI) is a heritable connective tissue disease characterized by bone fragility and fracture susceptibility. More than 95% of OI have dominant mutations in *COL1A1* or *COL1A2*, resulting in primary structural or quantitative defects of collagen type I. The recessive forms of OI are caused by mutations in *CRTAP*, *BMP1*, *CREB3L1*, *IFITM5*, *FKBP10*, *LEPRE1*, *PPIB*, *SP7*, *PLOD2*, *TMEM38B*, *SERPINF*, *SERPINH1*, *SEC24D*, *SPARC*, *WNT1* [1], which lead to defects of proteins interacting with collagen post-translationally. Recently, an X-linked form caused by mutations in *MBTPS2* was identified [2].

OI has been classified into several types according to clinical features and genetic alterations. Type XV OI, first described by Keupp in 2013, is caused by biallelic mutations in *WNT1* [3]. So far, at least 30 cases have been reported. In addition to bone fragility, many of them had neurological abnormalities [4–7]. Defects in Wnts or related proteins could lead to derangements in axonal pathways, including commissural axon tracts such as the corpus callosum [8]. Monoallelic mutations in *WNT1* cause early onset osteoporosis [9]. Genotype and phenotype correlation of *WNT1* mutations is not yet determined, requiring a larger cohort of patients.

Here, we describe two siblings with OI. One of them had brain malformations and global developmental delay, while the other had normal cognition. Mutation analysis identified a homozygous frameshift mutation in *WNT1*, which is the most 5′ change reported to date.

## Case presentation

**Fig. 1:**
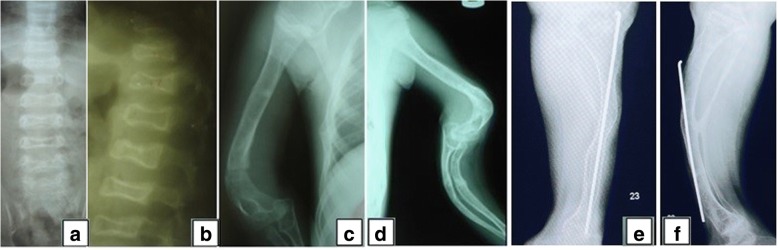
Radiological features of the proband. Imaging of the thoracic and lumbar spines at 14 months of age, **a** the antero-posterior and **b** lateral views revealed depressed multiple vertebrae. Figures **c-f** showed imaging at 14 years of age of upper extremities (**c-d**) and lower extremities (**e-f**) revealing deformities of humeri, left ulna and radius, right tibia and fibula, left tibia and fibula, respectively

A 14-year-old Thai girl was born via cesarean section due to premature rupture of the membrane with a birth weight of 2500 g. She is the first child of a consanguineous (second-degree relatives) couple. Both parents are healthy and have never had fractures. During her first year of life, she had delayed motor development and growth failure. At one year of age, she could not sit by herself and weighed 7.5 kg (< 3rd centile). She presented to our hospital at 14 months of age with fractures of both femora without a history of significant trauma. She was found to have ptosis of both eyes with normal teeth but no blue sclerae. She was small for her age. Her weight was 7.8 kg (3rd centile) and her length was 68 cm (< 3rd centile). Skeletal survey showed diffuse osteopenia, multiple healed fractures of the right humoral shaft, both tibiae and fibulae. Spine radiograph showed flattening and indentation of vertebral bodies (Fig. 1). A diagnosis of OI was made and intravenous bisphosphonate therapy (pamidronate 1 mg/kg/dose for 3 days) was initiated and given every 3 months. However, she sustained 1–2 long bone fractures per year from minor trauma. She required multiple corrective osteotomies to correct her deformities. At the last follow-up, she was 14 years old, weighing 20 kg. She could not walk due to her long bone deformity (Fig. 1). Remarkably, although she was in a special education class due to physical disabilities, her cognition was appropriate for age. She could talk fluently and do mathematics properly.

Prenatally, her younger sister was found to have a dilated fourth ventricle by an ultrasonography. She was born at term via cesarean section because of previous cesarean section and was diagnosed with hydrocephalus at birth. At 4 months of age, she had her first fracture without a history of a significant trauma, leading to a diagnosis of OI. Physical examination revealed a head circumference of 38 cm (> 95th centile) with a wide anterior fontanelle (3 × 3 cm.) and blue sclerae. She had global developmental delay (could not hold her head) and hypotonia. MRI of the brain demonstrated a large posterior fossa cyst connecting with the fourth ventricular system, moderate hydrocephalus, hypoplasia of cerebellar hemisphere with absence of cerebellar vermis, and hypoplasia of corpus collosum. She was also diagnosed with vesicoureteral reflux grade V and gastroesophageal reflux requiring tube feeding. The patient had multiple hospitalizations because of recurrent urinary tract infections and pneumonia. She expired at the age of one year.

**Fig. 2:**
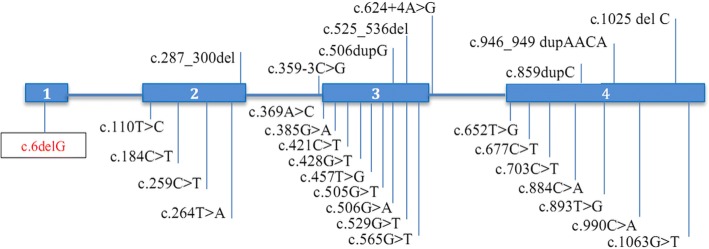
Reported mutations in *WNT1* [3–7, 9, 11–15] (solid bars represent coding exons of *WNT1*)

**Fig. 3:**
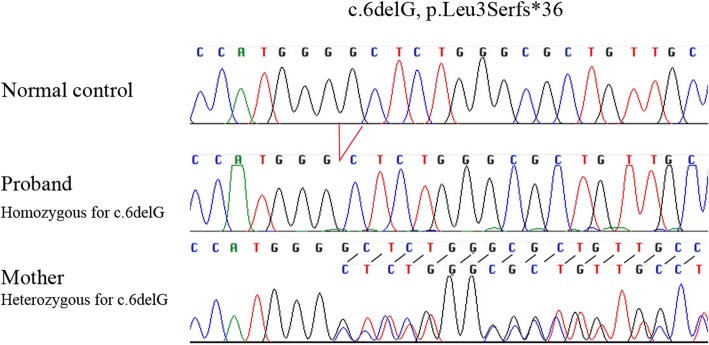
Mutation analysis. Sanger sequencing shows that the proband is homozygous while his mother is heterozygous for the *WNT1* c.6delG, p.Leu3Serfs*36 mutation

Sixteen known OI genes, *BMP1*, *COL1A1*, *COL1A2*, *CREB3L1*, *CRTAP*, *FKBP10*, *IFITM5*, *LEPRE1*, *PLOD2*, *PPIB*, *SERPINF1*, *SERPINH1*, *SP7*, *TMEM38B*, *WNT1*, and *MBTPS2*, were amplified from 200 ng of genomic DNA using the Truseq Custom Amplicon Sequencing kit (Illumina, San Diego, CA). 286 amplicons which covered all the 226 exons (28 kb) of the target genes were sequenced by Miseq (Illumina, San Diego, CA) using 2 × 250 paired-end reads. SNVs and Indels were detected by Miseq reporter software. The proband was found to harbor a homozygous mutation, c.6delG, p.Leu3Serfs*36 in *WNT1*. The mutation has never been reported in Human Gene Mutation Database (HGMD; http://www.hgmd.cf.ac.uk/ac/index.php) (Fig. 2). The mutation was subsequently confirmed by PCR-Sanger sequencing. Segregation analysis was performed by using primers, WNT1-E1F: GGT TGTTAAAGCCAGACTGC and WNT1-E1R: ACCAGCTCACTTACCACCAT. The results revealed that the patient was homozygous, while her mother was heterozygous for the mutation (Fig. 3).

## Discussion and conclusions

To better understand the clinical manifestations and natural history of patients with type XV OI, more patients and long-term follow-up are needed. Here, we report two siblings with a *WNT1* mutation. The older and younger sisters had their first fractures at 14 and four months of ages, respectively. Despite regular pamidronate administration for the older sister starting since then, at her last follow-up at 14 years of age, she sustained severe deformities of all extremities. The natural history of first fractures after birth, but severe bone deformities later in life is similar to the previous reported cases with *WNT1* mutations (Table 1: 55%, 10/18). Many OI patients with *COL1A1* or *COL1A2* mutations had fractures prenatally but did not have such severe bone deformities in their teens. This emphasizes the fact that onsets of fractures in patients with OI do not correlate with severities of final bone deformities.

**Table 1:** Features of patients with *WNT1* mutations (27 patients from 17 families)

Family-Case	Sex	Onset	Significant reported clinical features	Mutation	Ref.
I-1	F	1 y	Severe bone deformities, bilateral ptosis, normal cognition	c.6delG (p.Leu3Serfs*36)	This study
I-2	F	4 mo	Severe bone deformities, blue sclerae, DD, multiple brain anomalies	n/a	This study
II	M	n/a	Brain anomalies, unilateral ptosis, DD	c.184C > T (p.Gln62*) c.677C > T (p.Ser226Leu)	[4]
III	M	n/a	Thinning of the left temporal bone, ptosis, DD	c.259C > T (p.Gln87*) c.506dupG (p.Cys170Leufs*6)	[5]
IV	F	5 wk	Type 1 Chairi malformation of tonsillar descent, unilateral ptosis, autism	c.287_ 300delAGTTCCGGAATCGC (p.Gln96Profs*54)	[5]
V-1	M	n/a	Severe bone deformities	c.359-3C > G	[12]
V-2	F	n/a	Severe bone deformities	c.359-3C > G	[12]
VI	F	2 y	Mild bone deformities	c.369A > C (p.Glu123Asp) c.457 T > G (p.Cys153Gly)	[13]
VII-1	F	17 mo	Normal cognition	c.428G > T (p.Cys143Phe)	[9]
VII-2	M	2 wk	Normal cognition	c.428G > T (p.Cys143Phe)	[9]
VIII	M	1 mo	Bone deformity of lower extremities, normal cognition	c.525_536delCTTCGGCCGCCT (p.Phe176_Leu179del)	[15]
IX	F	2 mo	Severe bone deformities, blue sclerae, normal cognition	c.529G > T (p.Gly177Cys)	[3]
X	M	prenatal	Severe bone deformities, normal cognition	c.565G > T (p.Glu189*)	[3]
XI	M	7 mo	Severe bone deformities, normal cognition	c.624 + 4A > G	[3]
XII-1	M	3 mo	Severe bone deformities, normal cognition	c.859dupC (p.His287Profs*30)	[3]
XII-2	F	1 d	Severe bone deformities, blue sclerae, normal cognition	c.859dupC (p.His287Profs*30)	[3]
XII-3	M	prenatal	Severe bone deformities, blue sclerae, DD	c.859dupC (p.His287Profs*30)	[3]
XIII-1	F	1 mo	Normal cognition	c.884C > A (p.Ser295*)	[6]
XIII-2	F	prenatal	Hypoplasia of the left cerebellar hemisphere with short midbrain, Unilateral ptosis, severe DD	c.884C > A (p.Ser295*)	[6]
XIV-1	M	3 h	Severe bone deformities, severe DD	c.884C > A (p.Ser295*)	[5]
XIV-2	M	birth	Severe bone deformities, severe DD, multiple brain malformation	c.884C > A (p.Ser295*)	[5]
XV-1	F	2 y	Blue sclerae, normal cognition	c.893 T > G (p.Phe298Cys)	[5]
XV-2	M	1 y	Normal cognition	c.893 T > G (p.Phe298Cys)	[5]
XVI-1	F	3 d	Severe bone deformities, faint blue sclerae, normal cognition	c.893 T > G (p.Phe298Cys)	[3]
XVI-2	M	1 mo	Severe bone deformities, faint blue sclerae, normal cognition	c.893 T > G (p.Phe298Cys)	[3]
XVI-3	M	10 d	Severe bone deformities, blue sclerae, normal cognition	c.893 T > G (p.Phe298Cys)	[3]
XVII	M	birth	Severe bone deformities, dilated ventricles with cerebral atrophic changes, severe DD	c.990C > A (p.Cys330*)	[7]

*d* days, *DD* delayed development, *f* family, *F* female, *M* male, *mo* months, *n/a* not available, *NL* normal, *unilat* unilateral, *wk*. weeks, *y* years

Some other features of OI are inconsistent between our two patients. Blue sclerae were observed in only the younger sister, but not the proband. Previous reports found that blue sclerae could be observed in some cases with *WNT1* mutations (Table 1). Consistent with previous studies, neither of our patients had dentinogenesis imperfecta. Notably, ptosis, one of the most common clinical presentations of OI patients with *WNT1* mutations was only found in the proband. It is not usually found in other types of OI. Brain anomalies or intellectual disabilities were observed in 33% (9/27) of the previous reported cases (Table 1). Many patients have normal cognitive development. Remarkably, our proband had normal intellectual development, while her younger sister had severe brain anomalies, including hydrocephalus detected prenatally. This demonstrates a significant variability in neurological involvement between the affected siblings in this family and suggests that neurological abnormalities in *WNT1* mutations might be subject to modifier genes, epigenetics or non-genetic factors. There are two other families in the literature with significantly different neurological manifestations among the family members; one affected sib had normal intelligence and the other had severe brain anomalies or delayed development [5, 6]. Intrafamilial variable expression of this gene was substantiated.

Using Truseq Custom Amplicon sequencing, our proband was found to have an autosomal recessive form of OI caused by a homozygous truncating mutation in *WNT1*. Her sister’s blood sample could not be obtained. With similar recurrent bone fractures, we assumed that she harbored the same homozygous mutation. *WNT1* mutations were identified as a cause of malformations of midbrain and cerebellum in early brain development in mice long before it was identified as a cause of OI in humans [10]. In 2014, Swaying (*Wnt1*^*sw/sw*^) mice carrying *WNT1* mutations were found to have OI phenotypes including bone fragility and severe osteopenia [4]. The first report of a patient with a *WNT1* mutation was in 2013 [3]. As far as we know, the mutation identified in our patient is the first reported mutation in exon 1 and is the most 5′ truncating mutation (Fig. 2). The out-of-frame G deletion at the starting codon of *WNT1* is expected to result in a nonsense-mediated mRNA decay (NMD). There are no in-frame methionines after the frameshifting variant, so there would not be the possibility of another in-frame start site.

The genotype and phenotype correlation of *WNT1* mutations has not been established. The homozygous mutation found in our proband, which is a frameshift starting from the second codon, is expected to lead to a nonfunctional WNT1. She had normal cognition at the age of 14 years, indicating that humans without functional WNT1 could be intellectually normal.

In summary, we report two siblings with an autosomal recessive OI caused by the most 5′ homozygous mutation in *WNT1.* The findings exemplify intrafamilial variability in the neurological phenotype and suggest that *WNT1* may not be necessary for normal human cognitive development.

## Abbreviations

COL1A1: Collagen type I, alpha 1
COL1A2: Collagen, type I, alpha 2
HGMD: Human Gene Mutation Database
MRI: Magnetic resonance imaging
OI: Osteogenesis imperfecta
PCR: Polymerase chain reaction
WNT1: Wnt Family Member 1
